# A Recombinant Fragment of Human Surfactant Protein D induces Apoptosis in Pancreatic Cancer Cell Lines *via* Fas-Mediated Pathway

**DOI:** 10.3389/fimmu.2018.01126

**Published:** 2018-06-04

**Authors:** Anuvinder Kaur, Muhammad Suleman Riaz, Valarmathy Murugaiah, Praveen Mathews Varghese, Shiv K. Singh, Uday Kishore

**Affiliations:** ^1^Biosciences, College of Health and Life Sciences, Brunel University London, Uxbridge, United Kingdom; ^2^Department of Gastroenterology and Gastrointestinal Oncology, University Medical Center, Goettingen, Germany

**Keywords:** pancreatic cancer, innate immunity, surfactant protein D, apoptosis, immune surveillance

## Abstract

Human surfactant protein D (SP-D) is a potent innate immune molecule, which is emerging as a key molecule in the recognition and clearance of altered and non-self targets. Previous studies have shown that a recombinant fragment of human SP-D (rfhSP-D) induced apoptosis *via* p53-mediated apoptosis pathway in an eosinophilic leukemic cell line, AML14.3D10. Here, we report the ability of rfhSP-D to induce apoptosis *via* TNF-α/Fas-mediated pathway regardless of the p53 status in human pancreatic adenocarcinoma using Panc-1 (p53^mt^), MiaPaCa-2 (p53^mt^), and Capan-2 (p53^wt^) cell lines. Treatment of these cell lines with rfhSP-D for 24 h caused growth arrest in G1 cell cycle phase and triggered transcriptional upregulation of pro-apoptotic factors such as TNF-α and NF-κB. Translocation of NF-κB from the cytoplasm into the nucleus of pancreatic cancer cell lines was observed *via* immunofluorescence microscopy following treatment with rfhSP-D as compared to the untreated cells. The rfhSP-D treatment caused upregulation of pro-apoptotic marker Fas, as analyzed *via* qPCR and western blot, which then triggered caspase cascade, as evident from cleavage of caspase 8 and 3 analyzed *via* western blot at 48 h. The cell number following the rfhSP-D treatment was reduced in the order of Panc-1 (~67%) > MiaPaCa-2 (~60%) > Capan-2 (~35%). This study appears to suggest that rfhSP-D can potentially be used to therapeutically target pancreatic cancer cells irrespective of their p53 phenotype.

## Introduction

Human surfactant protein D (SP-D), a member of soluble C-type lectin family called Collectins, plays a vital role in linking the innate and adaptive immunity to protect against infection, allergy, and inflammation (1). Although its homeostatic role in lungs has been widely studied, its specific functions at extra-pulmonary tissues such as kidney, human trachea, brain, testis, heart, prostate, kidneys, and pancreas are poorly understood (1–3). SP-D deficiency in animal models has been shown to be associated with considerable pathophysiological consequences (4–6). SP-D gene knockout mice showed chronic inflammation and fibrosis due to accumulation of surfactant phospholipids in the lungs, monocytes infiltration, and activation of pro-inflammatory alveolar macrophages (5, 6). The absence of SP-D in children makes them more susceptible to frequent pneumonia as compared to SP-D sufficient children (7). *SFTPD* (SP-D gene) polymorphisms increase the susceptibility to chronic and infectious lung diseases (8), pneumococcal lung disease (9), emphysema (10), tuberculosis (11, 12), Crohn’s disease, and ulcerative colitis (12).

SP-D has been shown to be a potent innate immune molecule at pulmonary as well as extra-pulmonary mucosal surfaces by virtue of its ability to control inflammatory response and helper T cell polarization (3). The first clue came *via* a murine model of allergic hypersensitivity, when therapeutic treatment with a recombinant fragment of human SP-D (rfhSP-D) lowered peripheral and pulmonary eosinophilia, in addition to specific IgE levels and Th2 cytokines in the spleen (13, 14). It turned out that rfhSP-D selectively induced apoptosis in sensitized eosinophils derived from allergic patients (15). Using an eosinophilic cell line, AML14.3D10 (a model cell line for leukemia), it was established, *via* proteomics analysis, that apoptosis induction by rfhSP-D involved upregulation of p53 (16, 17). Another crucial study by Pandit et al. (18) revealed that rfhSP-D was able to induce apoptosis in activated human PBMCs, but not in resting, non-activated PBMCs. These studies, for the first time, raised the possibility that SP-D can have a function of immune surveillance against activated self and perhaps altered self. Recently, human lung adenocarcinoma cells (A549 cell line), when exogenously treated with SP-D, showed suppressed epidermal growth factor (EGF) signaling by reducing the EGF binding to EGFR, which subsequently reduced the cell proliferation, invasion, and migration of cancer cells (19).

Here, we set out to examine a possible pro-apoptotic role of SP-D in pancreatic cancer. Pancreatic cancer is the fourth leading cause of cancer-related mortality in the western world (20, 21) and its 5-year survival rate is ~5% (22). The poor prognosis has been attributed to the silent nature of the tumor in early stages, aggressive phenotype, surgical complications, and lack of targeted efficacious therapies (23). In this study, we show that rfhSP-D, composed of 8 Gly-X-Y repeats, homotrimeric neck and carbohydrate recognition domains (CRDs) (1), induces cell growth arrest in G1 phase and subsequent apoptosis in human pancreatic adenocarcinoma cells using Panc-1, MiaPaCa-2, and Capan-2 cell lines. The apoptosis induction appears to involve TNF-α, NF-κB, and Fas axis, revealing a p53 independent route of apoptosis induction in the p53 mutated Panc-1 and MiaPaCa-2 cell lines and p53-dependent apoptosis in p53 wild type Capan-2 cell line by rfhSP-D.

## Materials and Methods

### Cell Culture and Treatments

Human pancreatic cancer cells lines, Panc-1 (CRL-1469), MiaPaCa-2 (CRL-1420), and Capan-2 (HTB-80), were obtained from ATCC and used as an *in vitro* model in this study. All cell lines were cultured at 37°C under 5% v/v CO_2_ using DMEM-F12 media (Thermo Fisher) containing 10% v/v fetal calf serum with 2 mM l-glutamine, and penicillin (100 U/ml)/streptomycin (100 µg/ml) (Thermo Fisher) until 80–90% confluency was reached.

### Expression and Purification of rfhSP-D

Plasmid pUK-D1 (containing cDNA sequences for 8 Gly-X-Y repeats, neck, and CRD region of human SP-D), transformed into *Escherichia coli* BL21 (λDE3) pLysS (Invitrogen), was used to express rfhSP-D, as described earlier (15, 16). The expression cassette included a short stretch of eight N-terminal Gly–X–Y triplets with substitution of S for P in position 2 (residue 180), followed by the α-helical coiled-coil neck region (residues 203–235) and the globular CRD region (residues 236–355). Endotoxin levels were determined using the QCL-1000 Limulus amebocyte lysate system (Lonza) and the assay was found to be linear over a range of 0.1–1.0 EU/ml (10 EU = 1 ng of endotoxin). The amount of endotoxin levels were <4 pg/μg of the rfhSP-D. Full length native SP-D (FL-SP-D) was purified form lung washings of alveolar proteinosis patients using methods previously described by Strong et al. (24).

### Fluorescence Microscopy

All cell lines used in this study (Panc-1, MiaPaCa-2, and Capan-2) were grown on coverslips using 0.5 × 10^5^ cells overnight. Next day, cells were washed three times with PBS before being incubated with rfhSP-D (20 µg/ml) in a serum-free DMEM-F12 medium. For rfhSP-D and FL-SP-D binding analysis, the coverslips were incubated for 1 h with mouse anti-human SP-D (rfhSP-D) and rabbit anti-human SP-D (FL-SP-D) (MRC Immunochemistry Unit, Oxford; 1:200), followed by goat anti-mouse IgG H&L (Cy5) and Goat anti-Rabbit IgG H&L Alexa Fluor 488 (1:500; Abcam), respectively, and Hoechst (1:10,000; Thermo Fisher) for fluorescence microscopy analysis. For apoptosis analysis *via* fluorescence microscopy using an FITC annexin V apoptosis detection kit with propidium iodide (PI) (BioLegend), the cells were incubated with rfhSP-D (20 µg/ml) for 48 h. After 48 h, the cells were incubated with annexin V binding buffer containing FITC annexin V (1:200), PI (1:200), and Hoechst (1:10,000) for 15 min, and washed twice with PBS before mounting on the slides to visualize under a HF14 Leica DM4000 microscope.

### Flow Cytometry

Cell lines were plated in a 6-well plate (0.1 × 10^7^) and incubated with rfhSP-D (20 µg/ml), FL-SP-D (10 and 20 µg/ml), and an untreated control, for 24 and 48 h, followed by cell detachment using 5 mM EDTA, pH 8, and centrifugation at 1,200 × *g* for 5 min. For cell cycle analysis, the cells were fixed in 70% v/v ethanol for 30 min at 4°C, followed by PBS wash twice at 850 × *g*. The cells were then treated with ribonuclease (100 µg/ml) to ensure DNA staining without RNA contamination before staining with PI (50 µg/ml). 10,000 cells were then acquired for both treated and untreated samples and the PI histograms were plotted using the set markers within the analysis program of Novocyte Flow Cytometer. For apoptosis analysis *via* FACS, FITC annexin V apoptosis detection kit with PI (BioLegend) was used, as per manufacturer’s instructions. Compensation parameters were acquired using unstained, untreated FITC stained, and untreated PI stained cells.

### MTT Assay

MTT (3-[4,5-dimethylthiazol-2-yl]-2,5-diphenyltetrazolium bromide) (Thermo Fisher) assay was performed by incubating pancreatic cancer cells (0.1 × 10^5^) in a 96-well microtiter plate with rfhSP-D, FL-SP-D (10 and 20 µg/ml), and an untreated control in serum-free DMEM-F12 medium for 48 h, followed by incubation with 50 µg/µl MTT (5 mg/ml stock) per well for 4 h at 37°C. Majority of the media was removed leaving behind 25 µl per well, which was mixed thoroughly with 50 µl of dimethyl sulfoxide and incubated for another 10 min at 37°C. The absorbance was read at 570 nm using a plate reader.

### Western Blot

Cell lines (0.1 × 10^7^ cells) were seeded in a 6-well plate (Nunc) and incubated with rfhSP-D (20 µg/ml), together with an untreated control, in a serum-free DMEM-F12 medium. The cells were lysed within the wells using treatment buffer (50 mM Tris-HCl pH 6.8, 2% v/v β-mercaptoethanol, 2% v/v SDS, 0.1% w/v bromophenol blue, and 10% v/v glycerol) and transferred to pre-cooled microcentrifuge tubes followed by sonication for 15 s. The samples were heated at 100°C for 10 min and subjected to SDS-PAGE (12% w/v) for 90 min at 120 V. The SDS-PAGE separated proteins were then electrophoretically transferred onto a nitrocellulose membrane (Thermo Fisher) using an iBLOT (Thermo Fisher). The membrane was then blocked using 5% w/v dried milk powder (Sigma) in 100 ml PBS for 2 h on a rotatory shaker at room temperature. The membrane was incubated with rabbit anti-human caspase primary antibodies (anti-cleaved caspase 3; anti-cleaved caspase 8; Cell Signaling) at 4°C overnight, followed by incubation with secondary Goat anti-rabbit IgG HRP-conjugate (1:1,000; Promega) for 1 h at room temperature. The membrane was washed with PBST (PBS + 0.05% Tween 20) three times, 10 min each time. The color was developed using 3,3′-diaminobenzidine substrate kit (Thermo Fisher).

### Quantitative RT-PCR

Panc-1, MiaPaCa-2, and Capan-2 cells were incubated with and without rfhSP-D (20 µg/ml) for various time points. The cell pellet for each time-point was centrifuged and stored at −80°C. RNA was extracted using GenElute Mammalian Total RNA Purification Kit (Sigma-Aldrich, UK), as per manufacturer’s instructions, followed by treatment with DNase I (Sigma-Aldrich, UK). The absorbance at 260 and 260:280 nm ratio was used to determine the concentration and purity of total RNA, respectively, using NanoDrop 2000/2000c (Thermo-Fisher Scientific). Total RNA (2 µg) was used for cDNA synthesis using High Capacity RNA to cDNA Kit (Applied Biosystems). The forward and reverse primers used in this study were designed using the web based Basic Local Alignment Search Tool and Primer-BLAST (http://blast.ncbi.nlm.nih.gov/Blast.cgi) are given in Table 1.

**Table 1:** Target genes and terminal primers used in the qPCR analysis.

Target gene	Forward primer	Reverse primer
18S	5′-ATGGCCGTTCT TAGTTGGTG-3′	5′-CGCTGAGCCAG TCAGTGTAG-3′
Fas	5′-ACACTCACCAG CAACACCAA-3′	5′-TGCCACTGTTTC AGGATTTAA-3′
mTOR	5′-TGCCAACTATCT TCGGAACC-3′	5′-GCTCGCTTCACC TCAAATTC-3′
TNF-α	5′-GTATCGCCAGG AATTGTTGC-3′	5′-AGCCCATGTTGT AGCAAACC-3′
NF-κB	5′-TGAGGTACAGGC CCTCTGAT-3′	5′-GTATTTCAACCAC AGATGGCACT-3′
P53	5′-AGCACTGTCCAA CAACACCA-3′	5′-CTTCAGGTGGCT GGAGTGAG-3′

Relative mRNA expression was determined by qPCR reactions performed in triplicates consisting of 10 µl final volume per well [5 µl Power SYBR Green MasterMix (Applied Biosystems), 75 nM of forward and reverse primers, and 500 ng cDNA], using the 7900HT Fast Real-Time PCR System (Applied Biosystems). Samples were initially incubated at 50°C (2 min) and 95°C (10 min), followed by 40 cycles (each cycle for 15 s at 95°C and 1 min at 60°C) for amplification of the template. Human 18S rRNA, an endogenous control, was used to normalize the gene expression. Relative quantification (RQ) value and formula: RQ = 2^−ΔΔCt^ was used to calculate the relative expression of each target.

### Statistical Analysis

Graphs were made and statistically analyzed using Graphpad Prism 6.0 by applying an unpaired two-way ANOVA test. Significance of values is based on *p < 0.05, **p < 0.01, ***p < 0.001, ****p < 0.0001 between treated and untreated samples. Error bars represent the SD or SEM, as indicated in the figure legends.

## Results

### rfhSP-D Binds to a Range of Pancreatic Cell Lines

**Figure 1:**
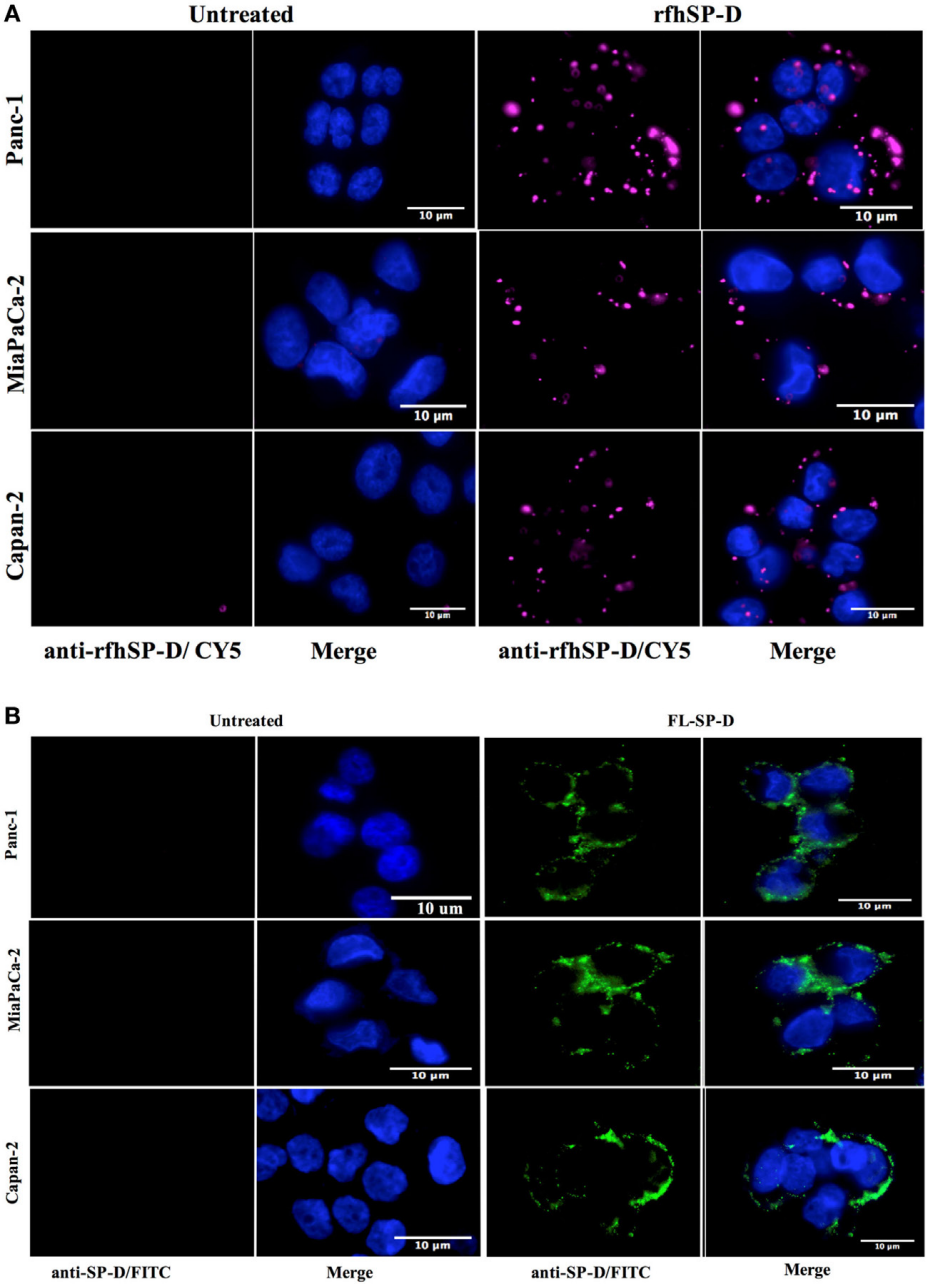
**(A)** Fluorescence microscopy showing binding of rfhSP-D and **(B)** FL-SP-D (10 µg/ml; 1 h incubation) to Panc-1, MiaPaCa-2, and Capan-2 cells. The nucleus of the cells was stained with Hoechst. Cells were probed with mouse anti-human SP-D/CY5 (rfhSP-D) and rabbit anti-human/FITC (FL-SP-D); the bound proteins are visible on the cell membrane in the treated cells. No CY5 or FITC fluorescence was detected in the untreated control cells.

The fluorescence microscopy analysis of rfhSP-D and FL-SP-D binding to Panc-1, MiaPaCa-2, and Capan-2 cells revealed its membrane localization following 1 h incubation at 4°C (Figure 1). The rfhSP-D probed with mouse anti-human SP-D-CY5 antibody and FL-SP-D probed with rabbit anti-human SP-D-FITC appeared evenly bound in clusters on the cell membrane, along with nucleus stained positively with Hoechst. All cell lines showed a similar rfhSP-D and FL-SP-D binding pattern. No CY5 or FITC fluorescence was detected in the untreated controls, probed with primary and secondary antibodies, for each cell line, suggesting the rfhSP-D and FL-SP-D binding observed in the treated cell lines was protein-specific.

### rfhSP-D Induces Cell Cycle Arrest in G1 Phase in Panc-1 and MiaPaCa-2

**Figure 2:**
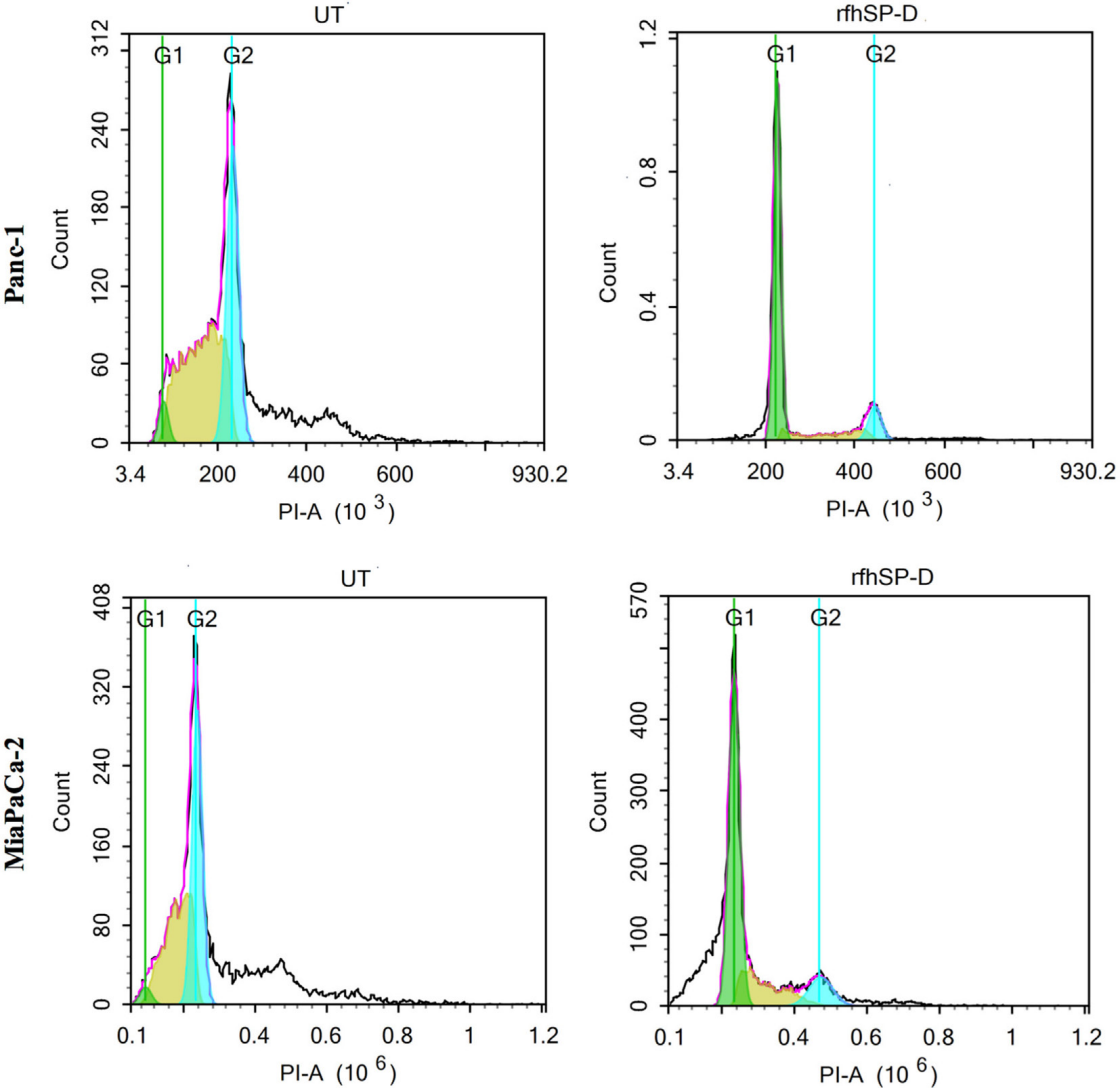
Cell cycle analysis following 24 h treatment of pancreatic cancer cell lines with rfhSP-D. Propidium iodide (PI) was used to stain DNA. PI histograms were plotted using set markers within the analysis program of Novocyte Flow cytometer. The rfhSP-D treated pancreatic cancer cells show arrest in G1 phase in the case of Panc-1 (G1 phase: 68%; S phase: 13%; G2 phase: 11%) and MiaPaCa-2 (G1 phase: ~50%; S phase: 17%; G2 phase: 10%) cell line at 24 h, whereas untreated Panc-1 cells (G1 phase: 3%; S phase: 42%; G2 phase: 32%) and MiaPaCa-2 cells (G1 phase: 2%; S phase: 32%; G2 phase: 33%) progressed to the next cell cycle phases.

Panc-1, MiaPaCa-2, and Capan-2 cell lines were individually treated with rfhSP-D for 24 h to assess whether rfhSP-D induced growth arrest. DNA binding dye, PI, was used to analyze the cell cycle for both treated and untreated cells *via* DNA quantitation. rfhSP-D induced inhibition of DNA synthesis in treated Panc-1 (68%) and MiaPaCa-2 (50%) in comparison to untreated Panc-1 (3%) and MiaPaCa-2 (2%) cells, respectively, as the cells were arrested in G1 phase (Figure 2). DNA synthesis was unaffected in the untreated cells for both cell lines since Panc-1 (43%) and MiaPaCa-2 (31%) were seen in S phase and Panc-1 (32%) and MiaPaCa-2 (33%) in the G2 phase of cell cycle. The growth arrest was, however, not seen in Capan-2 cell line following the rfhSP-D treatment (data not shown). Growth arrest at 24 h following rfhSP-D treatment prompted the determination of cell fate at a later time point; therefore, all cell lines were analyzed for likely apoptosis at 48 h.

### rfhSP-D Induces Apoptosis Induction in Pancreatic Cancer Cells by 48 h

**Figure 3:**
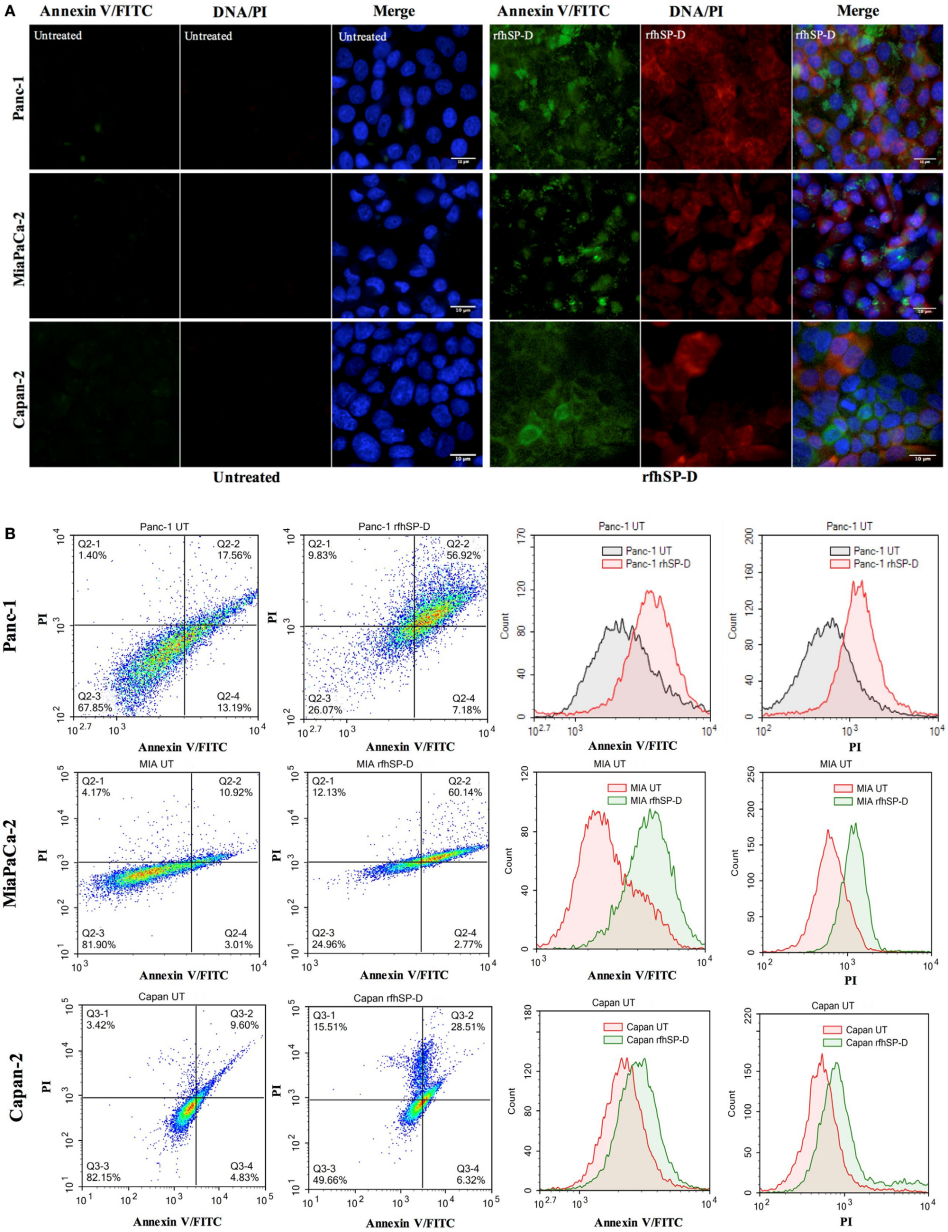

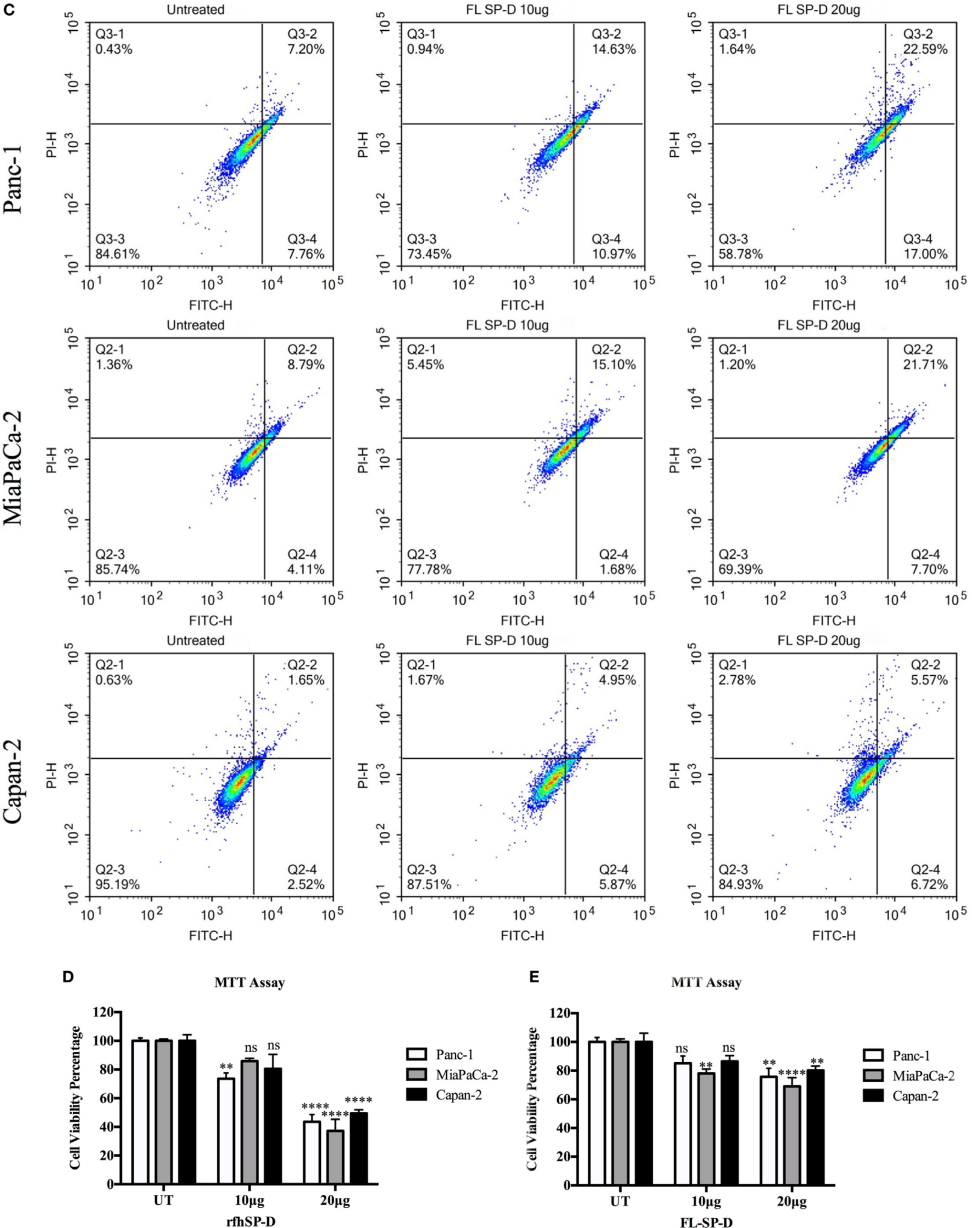
**(A)** Fluorescence microscopy to analyze apoptosis in pancreatic cancer cell lines following treatment with rfhSP-D. Cells were treated with rfhSP-D for 48 h and apoptosis was analyzed using an annexin V/propidium iodide (PI) staining kit. The cell membrane was positively stained for annexin V and the DNA staining is visible in the treated cells indicating that the cells underwent apoptosis turning the membrane inside out, thus making phosphatidylserine available for annexin V binding; due to the porous membrane, PI was taken in which stained the DNA of apoptotic cells. No such staining was seen in the untreated cells. The nucleus was stained with Hoechst for both treated and untreated cells. **(B,C)** Quantitative analysis of apoptosis using Flow Cytometer. Cells were treated with rfhSP-D or FL-SP-D for 48 h and apoptosis was analyzed using annexin V with PI kit. 10,000 cells were acquired and plotted for both annexin V/FITC and DNA/PI staining, which showed a shift in the fluorescence intensity of both FITC and PI between treated and untreated cells. Approximately 67% of Panc-1 cells, ~60% MiaPaCa-2 cells, and ~35% Capan-2 cells underwent apoptosis following rfhSP-D treatment and ~25% Panc-1 and MiaPaCa-2 cells following FL-SP-D treatment as compared to untreated cells. No significant difference was seen in Capan-2 cells following FL-SP-D treatment. **(D,E)** MTT assay to assess cell viability following treatment with rfhSP-D and FL-SP-D (10 and 20 µg/ml) and untreated for 48 h (±SEM, of three independent experiments). Cell numbers were reduced by approximately 70% in the rfhSP-D-treated Panc-1, 60% in MiaPaCa-2, and 45% in Capan-2 cells, as compared to untreated cells. Cell numbers were reduced by approximately 25% in the Panc-1 and MiaPaCa-2 and less than 10% in Capan-2 cells treated with FL-SP-D as compared to untreated cells. Significance was established using the unpaired two-way ANOVA test (**p < 0.01, ****p < 0.0001, ns: non-significant) (*n* = 3).

The qualitative apoptosis analysis of Panc-1, MiaPaCa-2, and Capan-2 treated with FL-SP-D or rfhSP-D for 48 h using immunofluorescence microscopy (Figure 3A) showed that the cell membrane was disoriented and the PI bound to DNA in the treated cells as compared to untreated cells, where no florescence was detected, indicating that cells were undergoing apoptosis at 48 h.

The flow cytometry analysis to quantify apoptosis showed significant reduction in the viable cell percentage of Panc-1, MiaPaCa-2, and Capan-2. The rfhSP-D induced apoptosis in ~67% of Panc-1 cells at 48 h, out of which, ~57% Panc-1 cells were both FITC and PI positive and ~7% were FITC alone positive, suggesting annexin V/FITC binding to phosphatidylserine, a cell membrane phospholipid, which is externalized during early apoptotic stage and the passage of PI, a DNA stain, through the porous cell membrane into the nucleus in order to intercalate the DNA. Approximately, 10% cells were PI alone positive, which suggested that these cells were either dead or in late apoptotic stage. The percentage of viable cells, i.e., unstained, in the untreated sample was significantly higher (70%) as compared to treated (26%) (Figure 3B). The rfhSP-D induced apoptosis in MiaPaCa-2 was ~60%. However, rfhSP-D induced apoptosis in Capan-2 (~35%) cell line, which was not as much as in Panc-1 and MiaPaCa-2 cell lines (Figure 3B). The treatment with FL-SP-D (20 µg/ml) for 48 h induced apoptosis in approximately 25% of Panc-1 and MiaPaCa-2 cell lines, and less than 10% in Capan-2 cell line. No significant difference was seen with FL-SP-D (10 µg/ml) treatment for 48 h in all the cell lines investigated in this study (Figure 3C).

The cell viability analysis *via* MTT assay following rfhSP-D treatment showed ~60% decrease in the cell viability of Panc-1 and MiaPaCa-2 and 45% in Capan-2 as compared to untreated (Figure 3D) and BSA (10 and 20 µg/ml; data not shown) controls. The cell viability analysis *via* MTT assay following FL-SP-D treatment also showed consistent reduction as seen in flow cytometer analysis (Figure 3E). Apoptosis was further confirmed by analyzing the activation of caspase to determine the pathway involved.

### rfhSP-D Activates Cleavage of Caspase 8 and 3

**Figure 4:**
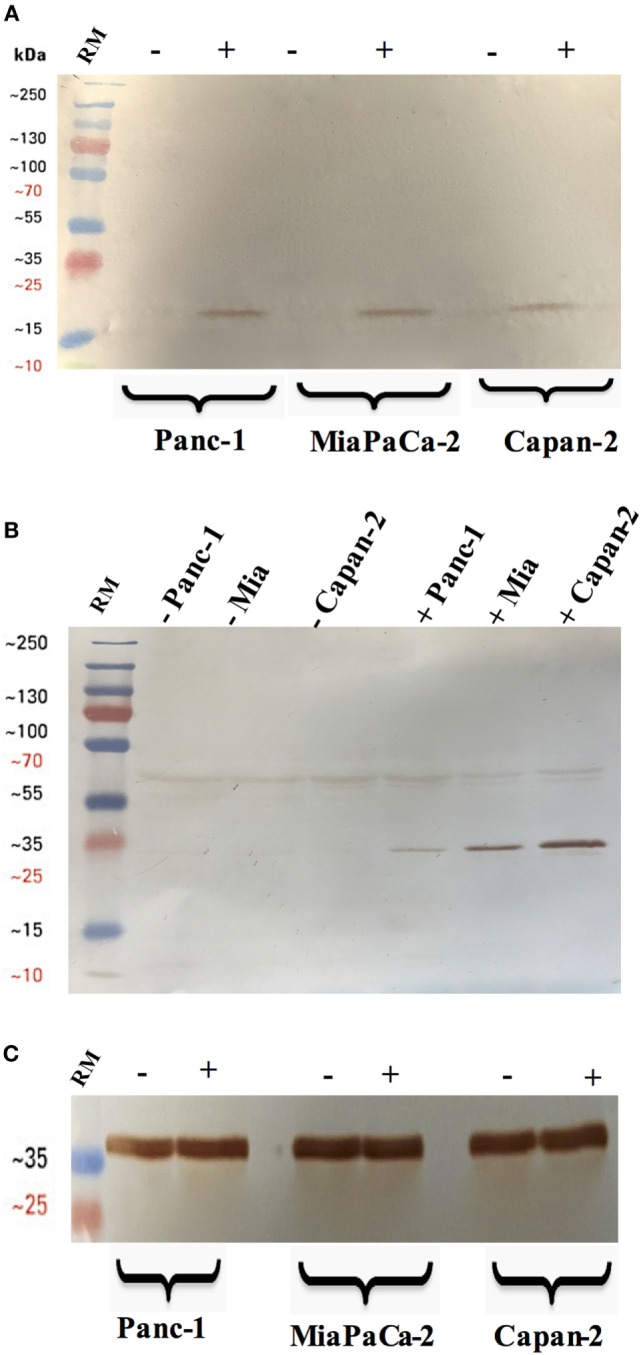
Cleavage of caspase 3 **(A)** and 8 **(B)** in pancreatic cancer cell lines following rfhSP-D treatment. Pancreatic cancer cell lines were analyzed for caspase 8 and 3 activation *via* western blot using anti-rabbit cleaved caspase 3 and 8 (1:1,000) at 4°C overnight, followed by incubation with secondary anti-rabbit IgG HRP-conjugate (1:1,000) for 1 h at room temperature. The membrane was washed with PBST (PBS + 0.05% Tween 20) three times, 10 min each between each step. The bands were developed using 3,3′-diaminobenzidine substrate kit. The cleaved caspase 3 and 8 were detected only in the rfhSP-D treated samples of all cell lines, whereas no bands appeared in the untreated cell samples. Full-length caspase 8 bands are visible around 43 kDa. **(C)** Anti-GAPDH was used as a loading control.

Western blot analysis revealed that caspase 8 and 3 were cleaved in all the cell lines following treatment with rfhSP-D for 48 h (Figure 4). The cleavage of caspase 3, however, was not seen in the untreated cells and faint bands appeared for caspase 8 in the untreated cells (Figure 4), which further confirmed that cell death occurred *via* apoptosis. Interestingly, although Capan-2 cell line appeared unaffected in terms of cell cycle arrest at 24 h; yet, the cleaved bands for caspase 8 and 3 were seen in Capan-2 treated cells too, which suggested that rfhSP-D can affect the cancer cells *via* multiple pathways. Caspase 9 was tested as a marker for intrinsic apoptosis pathway; however, no difference was noted between treated and untreated cells (data not shown). Therefore, gene expressions were assessed for pro-apoptotic genes such as Bax, an intrinsic pathway marker, and Fas, an extrinsic pathway marker, to further determine the apoptotic pathway.

### rfhSP-D Upregulates the Expression of Pro-Apoptotic Marker, Fas

**Figure 5:**
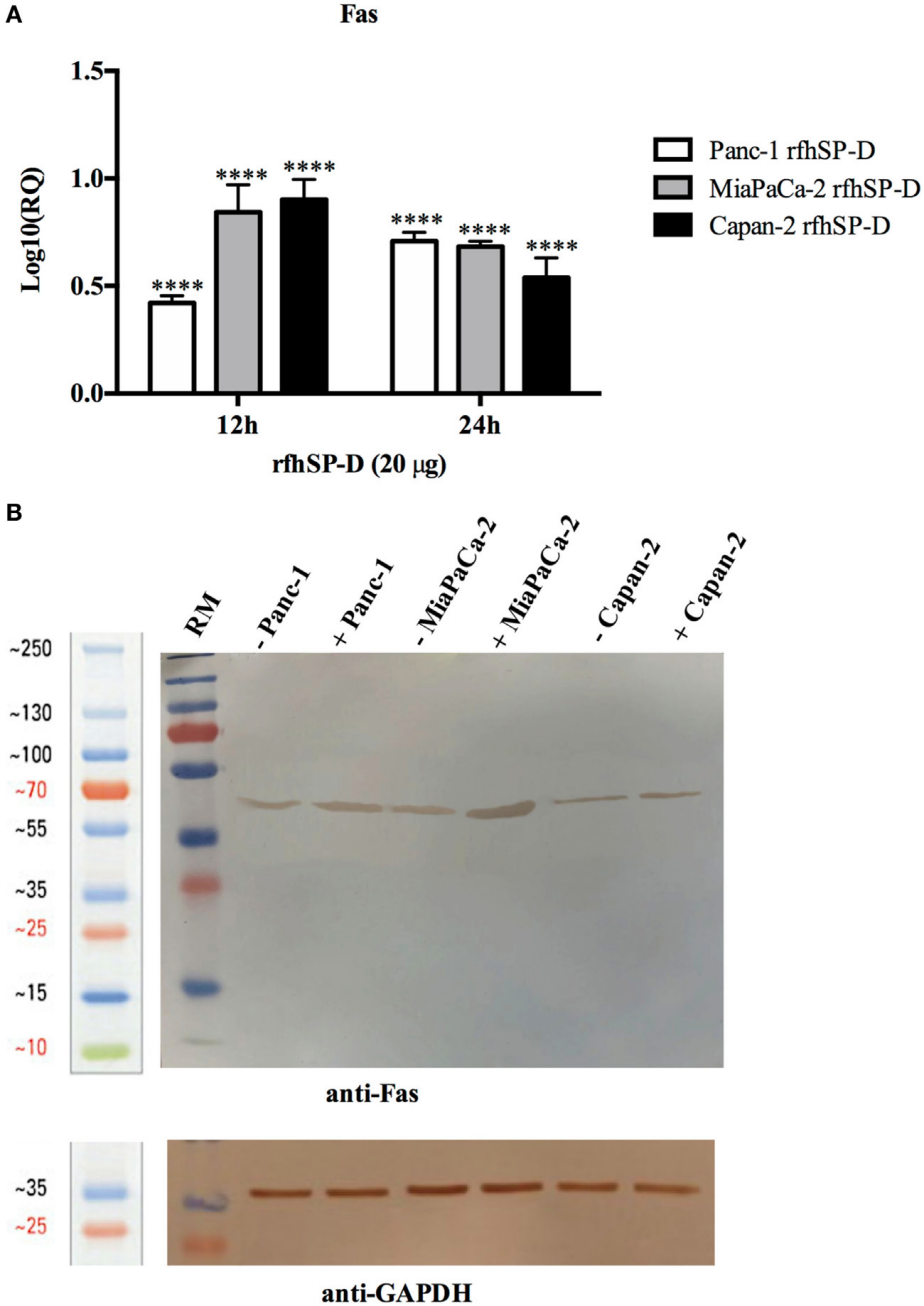
Relative quantification (RQ) of Fas mRNA expression in Panc-1, MiaPaCa-2, and Capan-2 cell lines treated with rfhSP-D (20 µg/ml) for 12 and 24 h. **(A)** Fas expression was upregulated in the treated samples at 12 and 24 h as compared to untreated cells. Significance was determined using the unpaired two-way ANOVA test (****p < 0.0001) (*n* = 3). **(B)** Fas expression *via* western blot analysis in pancreatic cell lines treated with rfhSP-D for 24 h using rabbit anti-human Fas (1:1,000) at 4°C overnight, followed by incubation with secondary anti-rabbit IgG HRP-conjugate (1:1,000) for 1 h at room temperature. The bands were developed using diaminobenzidine substrate kit. Fas expression at ~50 kDa was upregulated in the treated samples at 24 h for all cells as compared to untreated. Anti-GAPDH used as a loading control.

Human pancreatic cancer cells often escape apoptosis by downregulating apoptosis stimulators such as FasL/FasR (25), or pro-apoptotic proteins such as Bax (26). These pro-apoptotic genes, Bax and Fas, for time-points ranging from 2 to 24 h in all the cell lines, were analyzed. Bax was unaffected following the treatment with rfhSP-D in Panc-1 and MiaPaCa-2 cell lines at all time-points (data not shown), which, in addition to unaffected caspase 9, suggested that intrinsic pathway may not have been involved in causing the cell death in these cell lines. Fas expression was unaffected at earlier time-points up to 6 h (data not shown); however, it was upregulated at 12 and 24 h in Panc-1 (log_10_ ~0.5), MiaPaCa-2 (log_10_ ~1), and Capan-2 (log_10_ ~1) cell lines (Figure 5A), which indicated that apoptosis induction by rfhSP-D is likely to take place *via* the extrinsic pathway. Western blot analysis also showed upregulation of Fas at the protein level in rfhSP-D treated cells as compared to untreated cells (Figure 5B). Since TNF-α and NF-κB are crucial factors in the apoptotic pathway and they can regulate Fas expression (27), the effect of rfhSP-D on the gene expression of TNF-α and NF-κB as well as translocation of NF-κB from the cytoplasm to nucleus was investigated.

### rfhSP-D Upregulates p53 Expression in Capan-2 Cell Line

**Figure 6:**
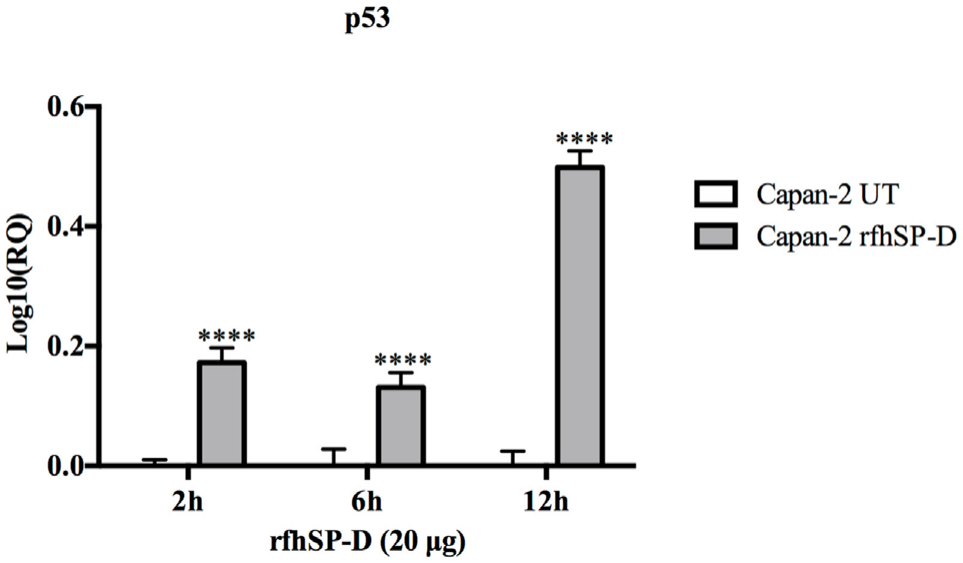
Relative quantification (RQ) of p53 mRNA expression in Capan-2 cell line treated with rfhSP-D (20 µg/ml) for 2, 6, and 12 h. p53 expression was significantly upregulated in the rfhSP-D-treated samples at 2, 6, and 12 h as compared to the untreated. Significance was determined using the unpaired two-way ANOVA test (****p < 0.0001) (*n* = 3).

The p53 transcript levels were measured by qPCR following the treatment with rfhSP-D at 2, 6, and 12 h in Capan-2 cells and compared with the p53 levels in untreated cells for each time-point. Interestingly, the levels of p53 were upregulated, most significantly at 12 h, which suggested that p53 may also have contributed to the apoptosis in Capan-2 cells (Figure 6).

### rfhSP-D Upregulates the Expression of TNF-α and Causes Nuclear Translocation of NF-κB

**Figure 7:**
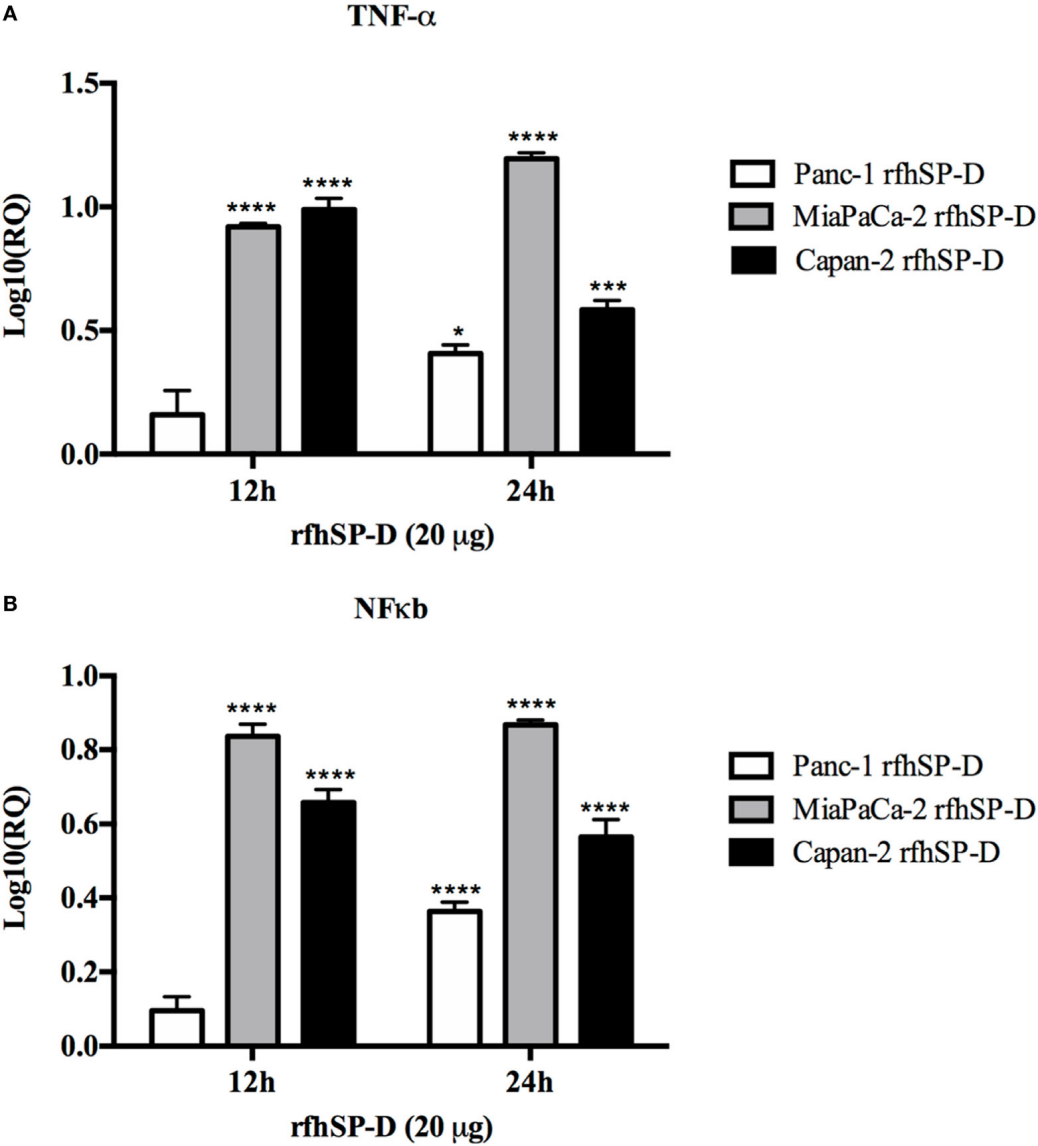

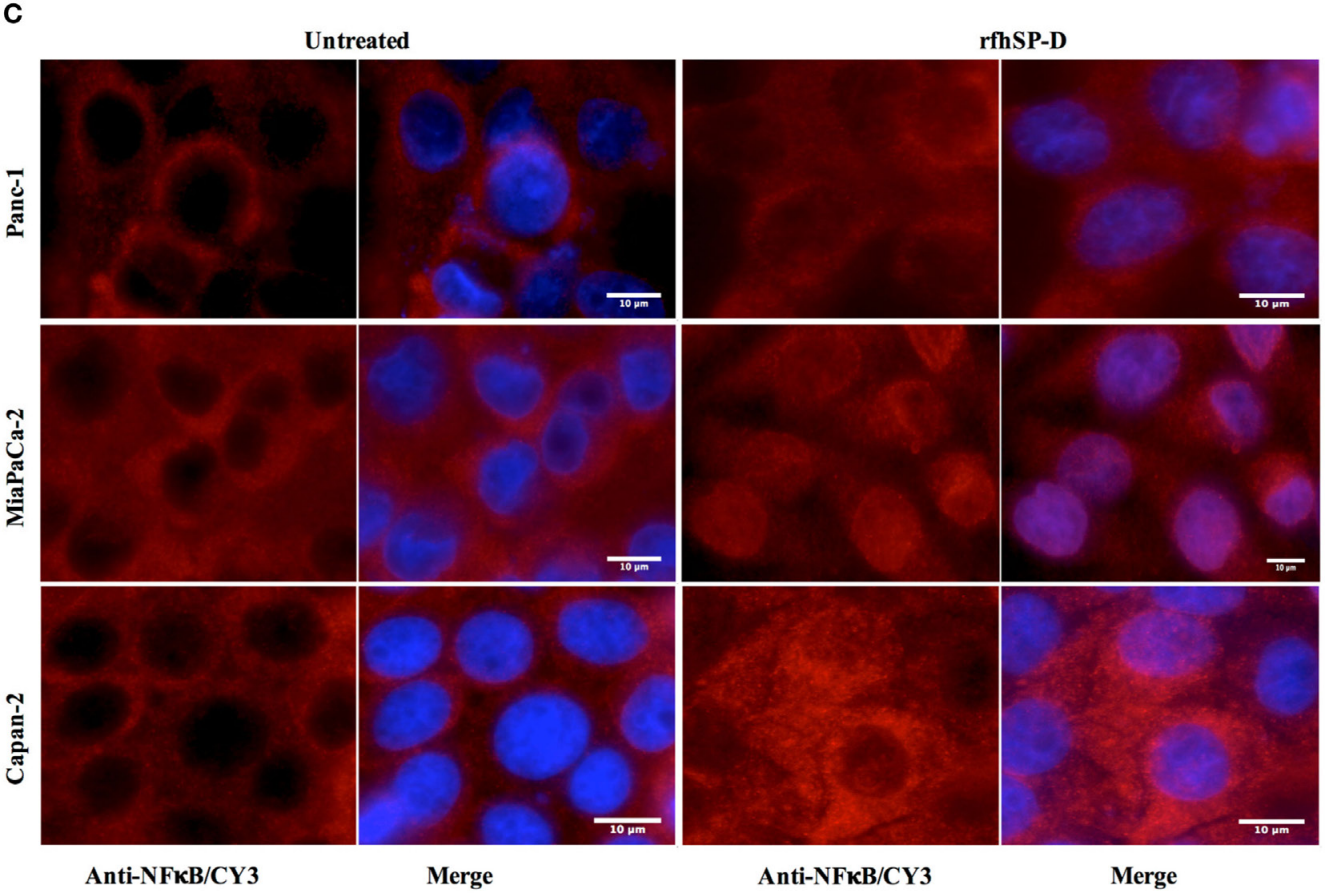
Relative quantification (RQ) comparisons of TNF-α **(A)** and NF-κB **(B)** mRNA expression in Panc-1, MiaPaCa-2, and Capan-2 cell lines treated with rfhSP-D (20 µg/ml) for 12 and 24 h. The transcriptional expressions of both TNF-α and NF-κB were upregulated in the treated samples at 12 and 24 h as compared to untreated. Significance was determined using the unpaired two-way ANOVA test (*p < 0.05, ***p < 0.001, ****p < 0.0001) (*n* = 3). **(C)** Immunofluorescence microscopy to determine the translocation of NF-κB into nucleus following rfhSP-D treatment. Anti-NF-κB stained positively in the nucleus of treated cells as compared to untreated in all cell lines at 24 h.

Following treatment with rfhSP-D, the analysis of TNF-α mRNA expression levels showed a significant upregulation in Panc-1 (log_10_ ~0.5), MiaPaCa-2 (log_10_ ~1), and Capan-2 (log_10_ ~1) at 12 and 24 h; however, no difference was observed at earlier time-points. Similar transcriptional upregulation was noted for NF-κB for Panc-1 (log_10_ ~0.4), MiaPaCa-2 (log_10_ ~0.8), and Capan-2 (log_10_ ~0.6) at 12 and 24 h (Figure 7A). Immunofluorescence microscopy of Panc-1, MiaPaCa-2, and Capan-2 cell lines showed that NF-κB was translocated to the nucleus at 24 h, which was not seen in the untreated cells (Figure 7B). This further confirmed that NF-κB could play a key role in deciding the apoptotic fate of the pancreatic cancer cells following the rfhSP-D treatment.

### rfhSP-D Downregulates the Survival Pathway, mTOR

**Figure 8:**
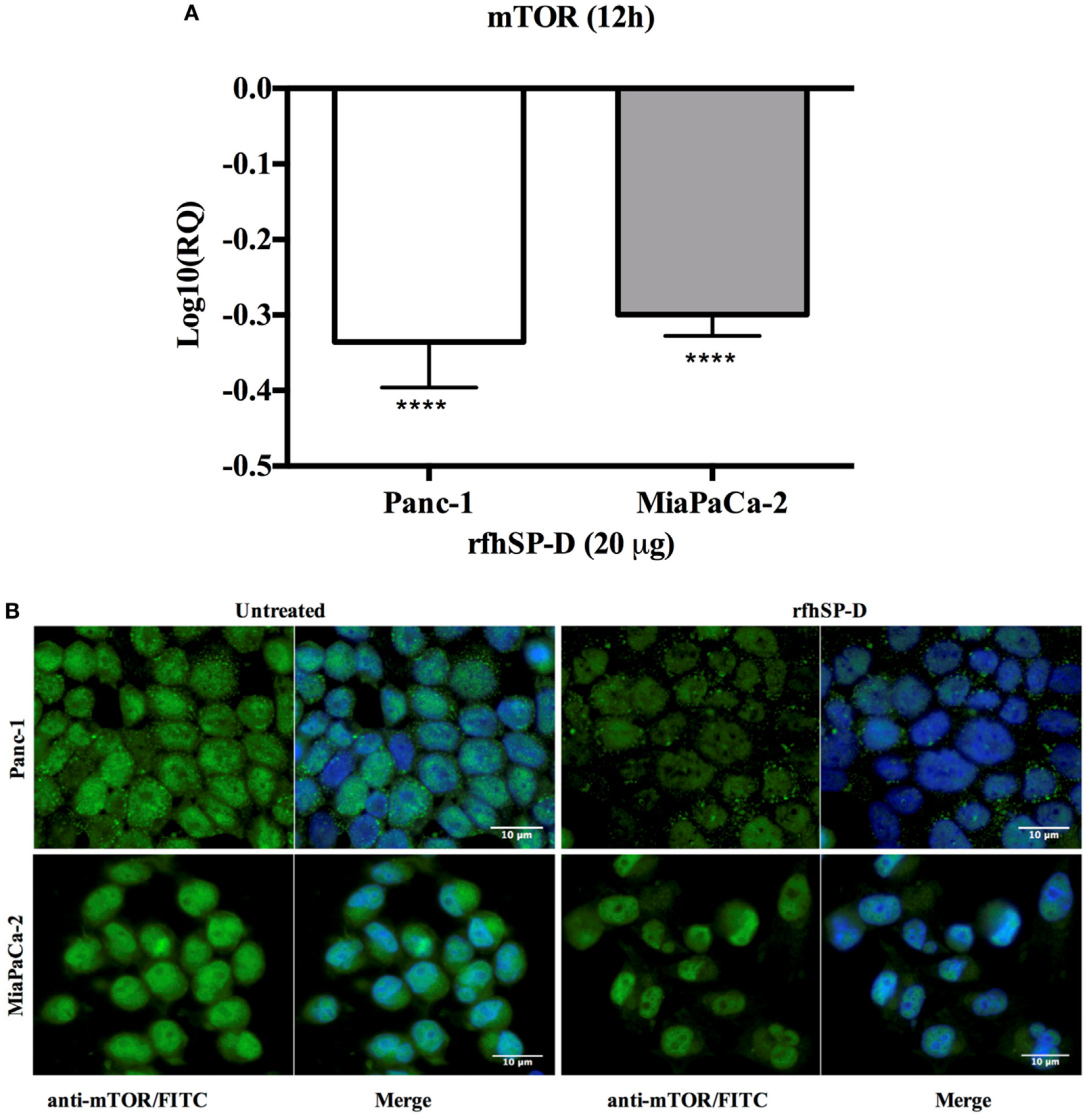
rfhSP-D downregulated the survival pathway, mTOR. **(A)** rfhSP-D treatment of Panc-1 and MiaPaCa-2 cells downregulated the mRNA expression of mTOR (****p < 0.0001). **(B)** Immunofluorescence microscopy showed reduced cytoplasmic levels of mTOR following treatment as compared to the untreated. Nuclear accumulation is clearly visible in the rfhSP-D treated cells.

The mTOR is often deregulated in the pancreatic cancer (28) and its activation is associated with poor prognosis (29). Upon treatment with rfhSP-D, mRNA expression of mTOR was downregulated in Panc-1 and MiaPaCa-2 cell line at 12 h (Figure 8A), however, no difference was seen in Capan-2 (data not shown). In addition, immunofluorescence analysis revealed that in comparison to the untreated cells, a significant decrease in the cytoplasmic levels and an increased accumulation of mTOR in the nucleus of MiaPaCa-2 cells was evident (Figure 8B), where it has been shown to be present in its inactive form (30).

## Discussion

In this study, we show that a recombinant fragment of human surfactant protein D (rfhSP-D) induces apoptosis in a range of pancreatic cancer cell lines. We show that rfhSP-D induces apoptosis regardless of p53 status using two p53 mutated, aggressive cell lines, Panc-1 (derived from head of the pancreas), MiaPaCa-2 (derived from the body and tail of the pancreas), and a p53 wild type, non-aggressive cell line, Capan-2 (derived from head of the pancreas) (31).

Following the treatment with rfhSP-D, Panc-1 and MiaPaCa-2 cells were arrested in G1 phase at 24 h, whereas untreated cells progressed to S and G2 phase. In addition, upregulation of Fas, an apoptosis stimulator, and pro-apoptotic TNF-α (and associated transcription factor, NF-κB) at 24 h was consistent with the cleavage of caspase 8 and 3 at 48 h. These findings indicated that cell death is likely to occur *via* TNF-α/Fas-mediated apoptosis pathway (32–34). The cell viability after 48 h of rfhSP-D treatment was reduced in the order of Panc-1 > MiaPaCa-2 > Capan-2, which coincided with the approximate growth arrested percentage of Panc-1 and MiaPaCa-2 at 24 h. Although Capan-2 cells were not arrested in the cell cycle, yet they underwent apoptosis at 48 h, which may be attributed to their increased sensitivity to Fas-mediated apoptosis as compared to other two cell lines (25) and upregulation of p53 transcripts following the treatment with rfhSP-D, as reported previously (16). Treatment with FL-SP-D induced apoptosis in approximately 25% of Panc-1 and MiaPaCa-2, compared to Panc-1 (~67%) > MiaPaCa-2 (~60%) > Capan-2 (~35) by rfhSP-D. This quantitative difference is likely to be due to difference in the molar ration of the two proteins at the same concentration.

Fas is a type I membrane protein that belongs to TNF superfamily (35, 36) that undergoes trimerization upon binding to its physiological ligand, FasL, to form a Fas-associated death domain protein (FADD) *via* its cytoplasmic domain (37, 38). It then activates downstream caspase cascade, which subsequently causes cleavage of caspase 3 as the terminal molecular event during apoptosis (39, 40). When the Panc-1, MiaPaCa-2, and Capan-2 cell lines were treated with rfhSP-D, Fas remained unaffected up to 12 h. Upregulation of Fas transcripts as well as protein was seen at 24 h, indicating that TNF-α (41) and NF-κB (33) might also be affected since they are well known to tightly regulate the Fas-mediated apoptosis pathway. TNF-α, another member of TNF superfamily, acts *via* TNFR2 to increase the susceptibility of the target cells to Fas-mediated death; in addition, it stimulates the downstream NF-κB signaling (42) by recruitment and activation of inhibitor of IκB kinases (IKK), which in turn enables its translocation to the nucleus where transcription of NF-κB-dependent genes such as Fas occurs (33, 43).

In this study, transcriptional levels of both NF-κB and TNF-α were upregulated at the same time-point as Fas, which was largely anticipated (33). In addition, the immunofluorescence microscopy revealed NF-κB translocation to nucleus at 24 h in the rfhSP-D-treated cells as compared to the untreated counterpart, which suggested that TNF-α induced canonical NF-κB pathway (44). NF-κB can regulate both pro- as well as anti-apoptotic genes, depending upon its canonical or non-canonical signaling (43, 44). Interestingly, canonical NF-κB has been shown to bind directly to the Fas promoter to facilitate cell death *via* Fas-mediated pathway (33). NF-κB plays an important role in deciding the cell fate as its canonical activation acts a transcription factor of Fas, which upon stimulation induces apoptosis signaling (32, 33). However, SP-D has been shown to regulate steady-state NF-κB activation in alveolar macrophages of SP-D deficient mice (45). Interestingly, SP-D has also been shown to trigger TNF-α production in human CCR2^+^ inflammatory monocytes (46). These studies present an interesting central role of SP-D and their interdependent regulation, which could be important in deciding the cell viability/apoptosis. Moreover, cleaved caspase 8 and 3 were seen at 48 h, whereas intrinsic apoptosis markers such as caspase 9 and Bax remained unaffected (27), in all rfhSP-D-treated pancreatic cancer cell lines as compared to untreated cells, which further confirmed the cell death *via* Fas-mediated pathway alone. In addition, mTOR pathway was downregulated following the treatment with rfhSP-D, which is crucial for cell survival and proliferation, and thus, to protect the cancer cells from apoptosis (47). These findings are also supported by studies such as targeting mTOR pathway using rapamycin (48), or its regulating component RICTOR knockdown (49), significantly reduces the pancreatic cancer cell growth. Interestingly, immunofluorescence microscopy showed that rfhSP-D causes nuclear accumulation of mTOR in the treated cells, which may have a transcriptional role. However, the nuclear versions do not form an intact mTORC1 required for regulatory signaling pathways (30).

rfhSP-D bound all the pancreatic cell lines tested in this study: Panc-1, MiaPaCa-2, and Capan-2 (Figure 1A). However, the putative SP-D receptor or the ligand on the pancreatic cancer cell surface is not yet known. Recently, an interaction between the CRD region of human SP-D and N-glycans of EGFR has been reported which led to downregulated EGF signaling in human lung adenocarcinoma, A549 cell line cells (19).

In conclusion, rfhSP-D upregulates pro-apoptotic factors such as TNF-α, NF-κB, and Fas to activate caspase cascade to induce apoptosis in pancreatic cancer cell lines, which needs further exploration in orthotropic murine models. Majority of the conventional anti-cancer therapies only target the rapidly proliferating cancer cells, therefore, new strategies involving immune molecules such as rfhSP-D that target the signaling pathways to reduce the cell growth merit further investigation as these would not only help eliminate the tumor but could also influence recurrence or migratory capacity of the tumor cells.

## Author Contributions

AK carried out most crucial experiments and was supported by MR, VM, and PV. SS provided ideas for crucial experiments and offered important reagents. AK wrote the first draft. UK led the study and helped with the manuscript preparation.

## Conflict of Interest Statement

The authors declare that the research was conducted in the absence of any commercial or financial relationships that could be construed as a potential conflict of interest.
